# Triglyceride-rich lipoproteins and cardiovascular diseases

**DOI:** 10.3389/fendo.2024.1409653

**Published:** 2024-05-31

**Authors:** Dandan Xu, Lin Xie, Cheng Cheng, Fei Xue, Chaonan Sun

**Affiliations:** ^1^ State Key Laboratory for Innovation and Transformation of Luobing Theory, Key Laboratory of Cardiovascular Remodeling and Function Research, Chinese Ministry of Education, Chinese National Health Commission and Chinese Academy of Medical Sciences, Department of Cardiology, Qilu Hospital of Shandong University, Jinan, China; ^2^ Department of Cardiology, Shengjing Hospital of China Medical University, Shenyang, Liaoning, China; ^3^ Department of Radiation Oncology, Cancer Hospital of China Medical University, Cancer Hospital of Dalian University of Technology, Liaoning Cancer Hospital and Institute, Shenyang, China

**Keywords:** atherogenic dyslipidemia, atherosclerosis, triglyceride-rich lipoproteins, triglyceride, lipolysis, apolipoproteins, lipoprotein lipase

## Abstract

The global prevalence of cardiovascular diseases (CVD) continues to rise steadily, making it a leading cause of mortality worldwide. Atherosclerosis (AS) serves as a primary driver of these conditions, commencing silently at an early age and culminating in adverse cardiovascular events that severely impact patients’ quality of life or lead to fatality. Dyslipidemia, particularly elevated levels of low-density lipoprotein cholesterol (LDL-C), plays a pivotal role in AS pathogenesis as an independent risk factor. Research indicates that abnormal LDL-C accumulation within arterial walls acts as a crucial trigger for atherosclerotic plaque formation. As the disease progresses, plaque accumulation may rupture or dislodge, resulting in thrombus formation and complete blood supply obstruction, ultimately causing myocardial infarction, cerebral infarction, and other common adverse cardiovascular events. Despite adequate pharmacologic therapy targeting LDL-C reduction, patients with cardiometabolic abnormalities remain at high risk for disease recurrence, highlighting the importance of addressing lipid risk factors beyond LDL-C. Recent attention has focused on the causal relationship between triglycerides, triglyceride-rich lipoproteins (TRLs), and their remnants in AS risk. Genetic, epidemiologic, and clinical studies suggest a causal relationship between TRLs and their remnants and the increased risk of AS, and this dyslipidemia may be an independent risk factor for adverse cardiovascular events. Particularly in patients with obesity, metabolic syndrome, diabetes, and chronic kidney disease, disordered TRLs and its remnants levels significantly increase the risk of atherosclerosis and cardiovascular disease development. Accumulation of over-synthesized TRLs in plasma, impaired function of enzymes involved in TRLs lipolysis, and impaired hepatic clearance of cholesterol-rich TRLs remnants can lead to arterial deposition of TRLs and its remnants, promoting foam cell formation and arterial wall inflammation. Therefore, understanding the pathogenesis of TRLs-induced AS and targeting it therapeutically could slow or impede AS progression, thereby reducing cardiovascular disease morbidity and mortality, particularly coronary atherosclerotic heart disease.

## 1 Introduction

Coronary atherosclerotic heart disease (CAD) stands as the predominant manifestation of organ disease stemming from atherosclerosis (AS), presenting a significant cardiovascular burden characterized by high morbidity, mortality, and low control rates. As reported in the China Cardiovascular Health and Disease Report 2020, the estimated CAD patient count in China approximates 11.39 million (1). And due to rural economic backwardness, poor basic medical care, low access to health services, and delays in the diagnosis and management of arteriosclerotic cardiovascular disease (ASCVD), the incidence of cardiovascular disease among rural residents is higher than that of urban residents (2). In 2018, China observed a continuing upward trend in resident CAD mortality rates since 2012, with urban residents experiencing mortality rates of 120.18/100,000 for CAD and 62.33/100,000 for acute myocardial infarction (AMI), while rural residents recorded rates of 128.24/100,000 for CAD and 78.47/100,000 for AMI. Furthermore, mortality rates for males surpassed those for females in both urban and rural areas (1). Throughout the early 21st century, fundamental research consistently unveiled new AS pathogenesis mechanisms and intervention targets, fostering the development, research, and application of novel anti-AS medications, including lipid-modulating drugs, anti-inflammatory agents, antiplatelet medications, angiotensin-converting enzyme inhibitors, and angiotensin receptor blockers. These pharmaceuticals have markedly decelerated AS progression, extended patient lifespans, and are extensively utilized in CAD treatment and prevention. Nevertheless, despite comprehensive pharmacologic and interventional therapy, patients with coronary artery disease confront a substantial residual risk of cardiovascular events. Hence, it remains crucial to delve further into novel AS pathogenesis mechanisms, identify new intervention targets, and implement targeted therapeutic approaches to effectively mitigate AS risk (3).

Atherosclerosis manifests as a chronic arterial wall disease culminating in clinical disease through either partial or complete blood vessel obstruction due to lumen narrowing or localized thrombosis. It stands as the foremost cause of vascular disease-related mortality globally (3). Characterized by plaque development in atherosclerotic arteries, originating from the intima and involving abnormal lipid accumulation, complex inflammatory reactions, necrosis, fibrous tissue proliferation, and calcium deposition, the gradual lesion progression to advanced disease stages may trigger secondary plaque hemorrhage, rupture, dislodgement, and local thrombus formation (4). Complete blood flow obstruction resulting from dislodged plaque or local thrombus formation may lead to myocardial infarction, cerebral infarction, and disabling peripheral arterial disease, posing severe threats to human health. Atherosclerosis development results from various risk factors acting on different stages, with smoking, obesity, dyslipidemia, hypertension, and diabetes mellitus among the principal risk factors, with abnormal lipid metabolism being paramount (5).

Observational epidemiological studies have conclusively demonstrated the significant and independent association of blood lipoproteins—namely, low-density lipoprotein cholesterol (LDL-C), high-density lipoprotein cholesterol (HDL-C), and lipoprotein(a)—with AS risk (6, 7). In recent years, the landscape of lipid-lowering drugs has expanded considerably, with statins, ezetimibe, and Proprotein Convertase Subtilisin/Kexin Type 9 (PCSK9) inhibitors serving as the primary agents. However, research indicates a residual risk of 65%-75% of cardiovascular events following statin therapy, and although combining statins with ezetimibe or PCSK9 inhibitors can further reduce the risk by 6% and 15%, respectively, a notable residual burden of vascular disease persists (8–10). This circumstance often correlates with elevated levels of triglyceride-rich lipoproteins (TRLs) and decreased levels of HDL-C, a dyslipidemia pattern increasingly prevalent due to rising rates of obesity, metabolic syndrome, and diabetes mellitus (11, 12). Over the past decade, an expanding body of demographic and genetic research concerning AS has consistently demonstrated the significant association of triglycerides, TRLs, and their remnants with AS risk (10, 11, 13, 14). Consequently, this review endeavors to scrutinize the role of TRLs in AS pathogenesis and identify novel therapeutic targets for AS treatment.

## 2 Synthesis and transport of triglyceride and TRLs

As a vital energy source for cellular metabolism, TG synthesis and transport are subject to intricate and precise regulation by multiple mechanisms *in vivo* (15). There are two main sources of plasma triglycerides, exogenous TG synthesized by intestinal epithelial cells from the absorption of digested dietary fat and endogenous TG synthesized by hepatic uptake and utilization of circulating free fatty acids (FFA) (16, 17). Exogenous TG can be assembled with other lipids in intestinal cells for CM conversion. While the liver acquires circulating FFA through various pathways, including the uptake of chylomicron residues, *de novo* lipogenesis (DNL) where glucose and other non-lipid precursors are converted to fatty acids, uptake of non-esterified fatty acids in plasma, and fatty acids released from hepatocyte lipid droplets, subsequently processed and assembled for very low density lipoprotein (VLDL) conversion (15, 18, 19). TRLs represent gut-derived chylomicron (CM) and liver-produced VLDL, and TRLs remnants are the lipolysis products of CM and VLDL (19, 20).

Apolipoproteins (Apo) function as carriers facilitating lipid translocation and mediating lipid metabolism through interactions with enzymes and cellular receptors (21). ApoB stands as the principal structural protein of TRLs, existing in two forms—ApoB100 and ApoB48—essential for intracellular assembly and VLDL and CM secretion (22, 23). ApoB100, synthesized by ribosomes attached to the rough endoplasmic reticulum (ER) of hepatocytes, forms very low-density lipoprotein precursors (pre-VLDL) in the ER lumen, subsequently converted to VLDL2 and further processed into highly atherogenic VLDL1 (22–24). ApoB48, produced in intestinal cells, participates in CM assembly and secretion (23). Dietary triglycerides undergo hydrolysis by digestive enzymes, yielding triglycerides and fatty acids absorbed by enterocyte parietal membranes, forming chylomicron precursors (pre-CMs) with apoB48 in the ER, further processed into mature CMs in the Golgi apparatus (25).

**Figure 1:**
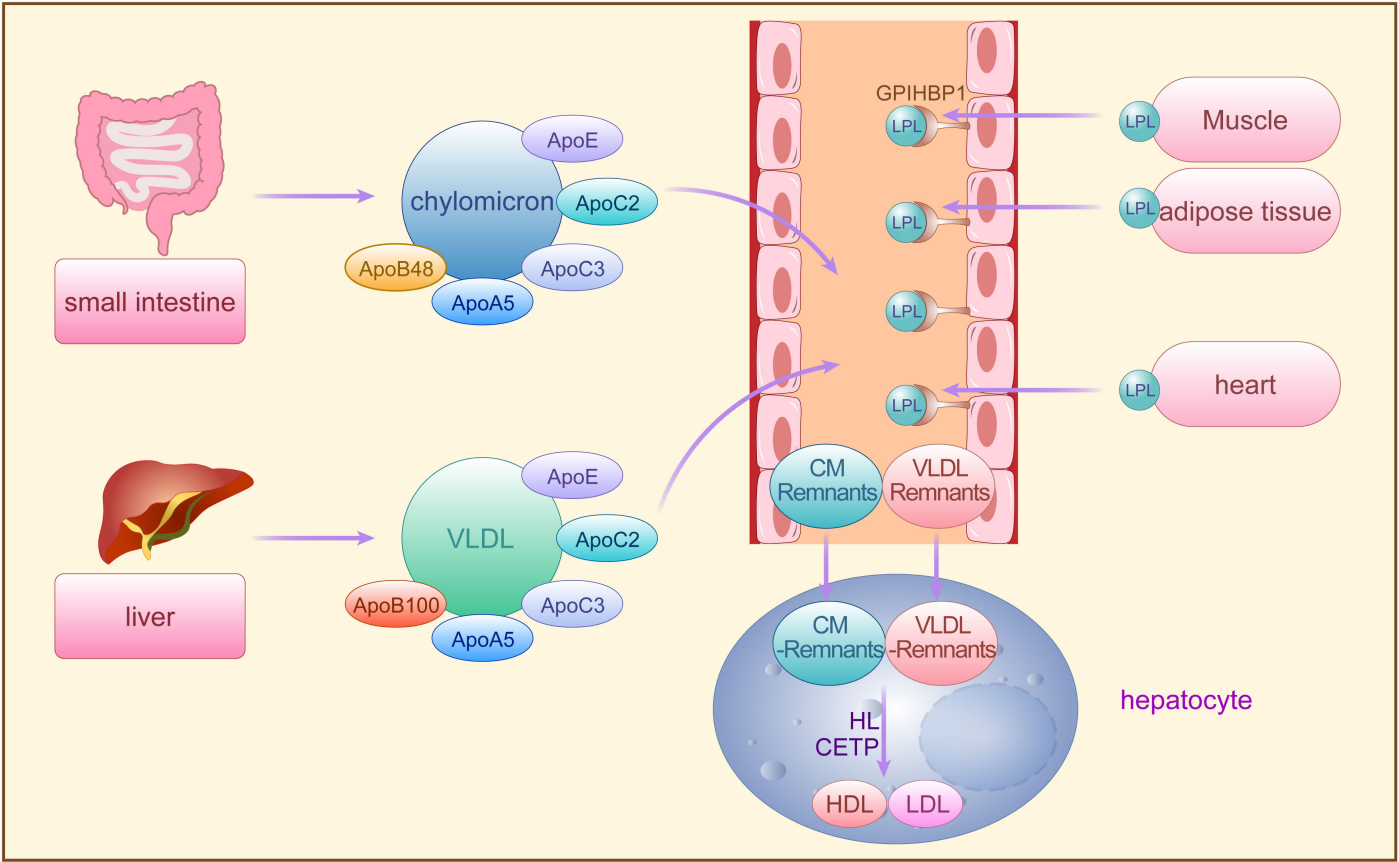
The synthesis and metabolism of TRLs. Exogenous TG synthesized by small intestine and endogenous TG synthesized by the liver are secreted into the bloodstream by CM and VLDL, requiring assembly with apoB48, apoB100, and other apolipoproteins, respectively, and then hydrolyzed by LPL secreted from adipose tissues storage, muscle tissues and heart. The CM residual particles generated during this period are cleared by the liver, while the VLDL remnants are further converted to LDL and HDL by HL and CETP. TG, triglycerides; CM, chylomicron; VLDL, very low-density lipoprotein; A po, apolipoproteins; LPL, lipoprotein lipase; FFA, free fatty acids; LDL, low-density lipoprotein; HL, hepatic lipase; CETP, cholesterol ester transfer protein; GPIHBP1, glycosylphosphatidylinositol-anchored HDL binding protein 1, an endothelial cell protein that shuttles LPL to the capillary lumen.

Triglycerides are predominantly transported in blood by VLDL and CM, requiring assembly with ApoB100, ApoC, ApoE, phospholipids, and cholesterol in hepatocytes for secretion into the bloodstream (26, 27). Dietary fatty acids, absorbed from the small intestine, are reassembled into TG, forming CMs with ApoB48, ApoC, ApoAI, ApoAIV, phospholipids, and cholesterol before secretion into lymphatic vessels and subsequently into plasma as triglyceride-rich chylomicrons. While over 70% of fatty acids are stored in adipose tissue, the remainder is absorbed by the liver (26, 27). In the fasting state, VLDL originating from the liver carries over 90% of blood TGs to peripheral tissues for utilization, while CM transports dietary fats, excluding short-chain fatty acids, to peripheral tissues (28) (**Figure 1**).

## 3 Metabolism of TG and TRLs

Lipoprotein lipase (LPL), situated on endothelial cell surfaces, is a pivotal enzyme engaged in the hydrolysis of CM-TG and VLDL-TG in peripheral tissues (29). TRLs upon entering circulation can acquire apolipoprotein CII (ApoCII) by exchanging apolipoproteins other than ApoB with other lipoprotein fractions. ApoCII facilitates LPL-mediated hydrolysis of TRLs-TG, releasing FFA for storage in adipose tissues or utilization by muscle tissues (29, 30). Initially, LPL-mediated TG lipolysis removes CM and VLDL, generating residual CM particles cleared by the liver, while VLDL remnants are further processed to LDL particles by hepatic lipase (HL) and cholesterol ester transfer protein (CETP) (29, 31). CETP mediates the exchange of TG and cholesterol esters (CE) between TRLs and high-density lipoproteins (HDL), transferring TG from TRLs to HDL and CE from HDL to TRLs (12, 32). Decreased TG and increased CE levels in TRLs lead to structural transformation into small, dense particles termed TRLs remnants (33). With the decrease of TG and the increase of CE in VLDL, VLDL undergoes the transformation of VLDL → VLDL remnant → IDL → LDL, and its diameter decreases sequentially (IDL: 25-35 nm; LDL: 18-25 nm; VLDL remnant: between IDL and VLDL). Approximately 25%-75% of VLDL is converted to LDL via this process (34), while the remaining VLDL remnants, along with CM remnants, may accumulate in plasma and continue to acquire CE from HDL until cleared by the liver (35). However, when fasting TG levels exceed 1.2 mmol/L, TRLs and TRLs remnants accumulate in plasma, with levels surpassing 1.7 mmol/L leading to accumulation exceeding clearance capacity. Remnants failing timely conversion to LDL persist in circulation, significantly augmenting TRLs potential contribution to AS pathology (35) (**Figure 1**).

## 4 Mechanisms by TRLs remnants promote AS lesions

In recent years, a plethora of genetic, epidemiological, molecular biological, and clinical investigations have unveiled a robust association between serum TG and TRLs levels and the residual risk of ASCVD. Efficient triglyceride transport *in vivo* ensures rapid clearance of TRLs, maintaining low plasma TRLs levels. Conversely, when plasma TG accumulates excessively or genetic variations diminish LPL activity, impaired TG transport efficiency ensues, fostering abnormal accumulation of circulating TRLs and its remnants within arterial walls, disrupting vascular homeostasis, and precipitating AS development (19, 35). Furthermore, plasma TRLs accumulation increases plasma viscosity, elevates fibrinogen expression, and decreases tissue-type fibrinogen activator activity, promoting platelet aggregation and thrombosis (36). Positive correlations between TG and TRLs levels and plaque lesions and vascular inflammation have been documented in low- and intermediate-risk ASCVD populations with fasting serum TG levels exceeding 1.7 mmol/L (37). Notably, a study involving 73,513 individuals from Denmark revealed a 2.8-fold increase in ASCVD risk for every 1 mmol/L elevation in TRLs levels (38).

### 4.1 TRLs promotes AS by subendothelial accumulation

Currently, it is widely acknowledged that TRLs primarily promote AS through the cholesterol content in their remnants, potentially exerting a greater impact on AS development compared to LDL-C, a well-established AS risk factor (39). This is attributed to the elevated cholesterol content in TRLs remnants, facilitated by LPL-mediated rapid clearance of TG and CETP-mediated exchange of TG for cholesterol with HDL. Consequently, TRLs remnants contain higher cholesterol levels compared to *de novo* CM or VLDL(29). Moreover, hypertriglyceridemia disrupts the transfer of ApoCIII to HDL during normal TRLs catabolism, while also increasing VLDL-associated ApoCIII production in the liver (33, 39). Elevated levels of ApoCIII inhibit LPL activity and hepatic uptake of VLDL remnants, prolonging TRLs remnant retention in plasma and enhancing cholesterol binding (33). Thus, TRLs remnants, including non-converted VLDL remnants and CM remnants, may contain 5-20 times more cholesterol than LDL-C. Although TRLs remnants quickly traverse the endothelial layer, they exhibit delayed efflux, rendering them more prone to subendothelial accumulation (35). Additionally, unlike LDL-C, TRLs remnants can be phagocytosed by macrophages without requiring oxidative modification or enzymatic processing, thereby facilitating foam cell formation and plaque progression. This distinct mechanism further underscores the significant contribution of TRLs remnants to AS pathogenesis (40).

ApoB emerges as a pivotal player in the proatherogenic cascade. By interacting with proteoglycans in the extracellular matrix, ApoB facilitates the retention and aggregation of surplus cholesterol-rich lipoproteins within the subendothelium of the arterial wall—an essential initial step in AS formation (41, 42). Lipoproteins containing ApoB with a diameter less than 70 nm, including CM remnants, VLDL, VLDL remnants, IDL, and LDL, can efficiently traverse the intact endothelium, allowing products from TRLs metabolism, except *de novo* CM, to accumulate beneath the vascular endothelium (43). Even minimal amounts of ApoB entering the arterial wall can instigate a selective retention mechanism, driving local accumulation of TRLs remnants, thus fostering AS progression.

TRLs and their remnants sequestered within the arterial wall undergo various modifications, such as oxidation, enzymatic or non-enzymatic cleavage, and aggregation, rendering them pro-inflammatory. This prompts vascular endothelium activation, leading to chemokine secretion and adhesion molecule expression, facilitating monocyte attraction and binding. Early research indicates that postprandial production of TRLs and their remnants is proinflammatory, particularly in hyperlipidemic patients (44, 45). Furthermore, in a pro-inflammatory milieu, TRLs and their remnants accumulate in the subendothelial layer, directly interacting with endothelial cells within the lumen (36). TRLs and their remnants may promote thrombosis by influencing endothelial thromboplastin status, and cholesterol in TRLs remnants accelerates endothelial progenitor cell (EPC) senescence, thereby inducing endothelial dysfunction (46, 47).

Subsequently, monocytes migrate from vascular endothelial cells to the subendothelial space, where they differentiate into macrophages, phagocytosing accumulated lipoproteins and forming foam cells. While this immune response initially serves a protective function, excessive lipoprotein accumulation surpassing macrophage clearance capacity results in cholesterol-rich foam cell accumulation, fostering plaque formation (48, 49). As the plaque evolves, macrophages and foam cells secrete additional extracellular matrix, further promoting TRLs and their remnants retention. Concurrently, the chronic inflammatory response continually recruits monocytes, T cells, mast cells, and other immune cells to the lesion site, exacerbating lesion expansion. In advanced stages, over-accumulated residual lipoproteins induce endoplasmic reticulum stress in macrophages, triggering specific apoptotic mechanisms and subsequent macrophage apoptosis (50). Apoptotic macrophages release TRLs remnants and other inflammatory debris, forming necrotic cores within plaques termed “fragile plaques,” susceptible to rupture and thrombus formation, precipitating acute cardiovascular events (51).

TRLs accumulation in the arterial wall stimulates smooth muscle cells (SMCs) proliferation and inflammation by activating genes involved in SMC proliferation and inflammation regulation (52). Subsequently, inflammation-activated VSMCs undergo transition to less differentiated macrophage-like cells, amplifying the wall’s existing inflammatory response by secreting various pro-inflammatory mediators, chemokines, and adhesion factors, thereby promoting AS progression (53). Furthermore, studies have demonstrated that triglyceride-rich lipoprotein remnants can stimulate the expression of monocyte chemotactic protein-1 (MCP-1) in VSMCs in a p38MAPK-dependent manner, promoting monocyte migration to the vascular wall and accelerating AS progression (54, 55).

**Figure 2:**
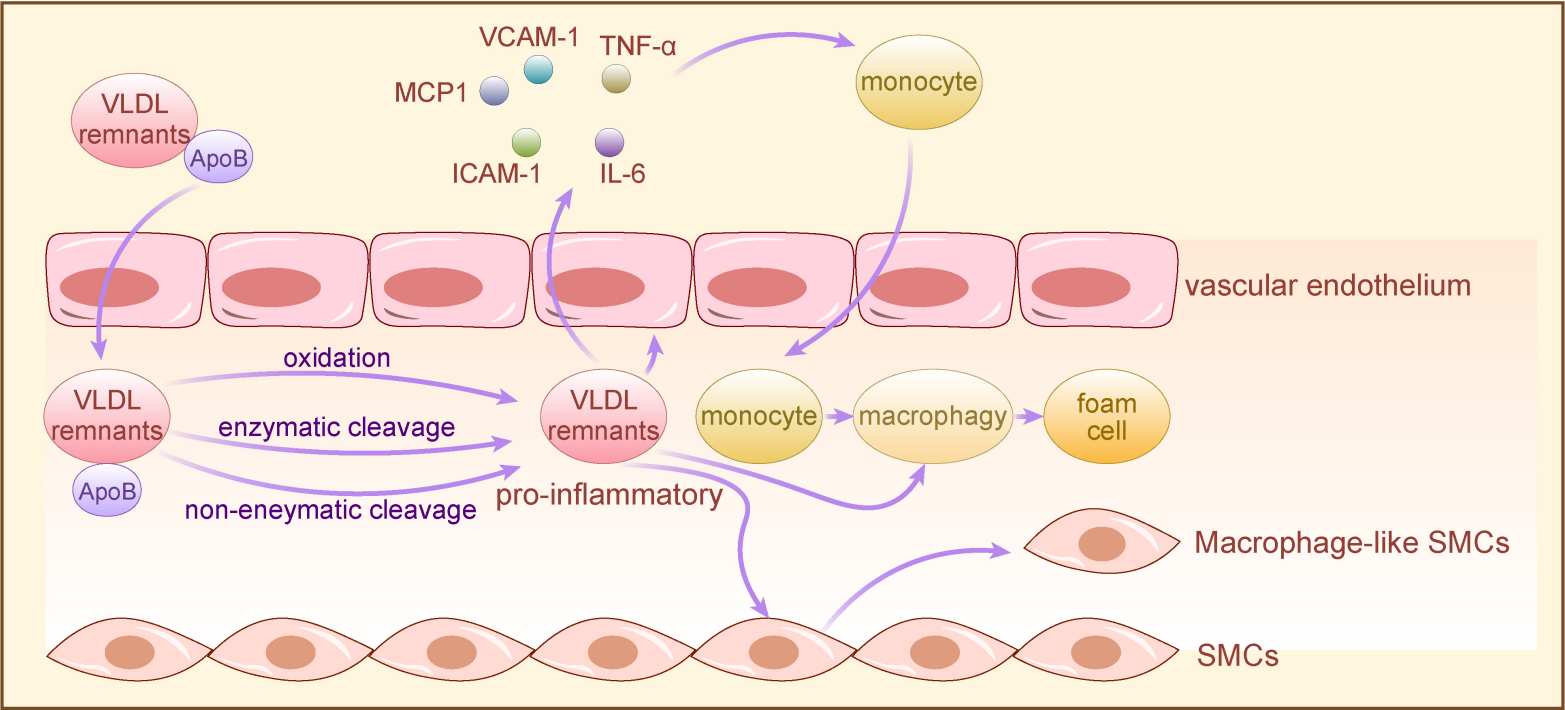
The mechanism of TRLs for AS development. TRLs and their remnants with long-term retention in the arterial wall are proinflammatory after oxidative, enzymatic or nonenzymatic cleavage modifications, and directly activate vascular endothelium to secrete chemokines and express adhesion molecules, leading to monocytes recruitment. Subsequently, monocytes differentiate into macrophages in vascular subendothelial space, co-phagocytizing lipoproteins with macrophages directly activated by pro-inflammatory TRLs to form foam cells. Moreover, pro-inflammatory TRLs induce VSMCs to convert to macrophage-like cells, exacerbating the wall’s pre-existing inflammatory response through secreting a variety of proinflammatory cytokines, thereby promoting AS. TRLs, triglyceride-rich lipoproteins; VSMCs, vascular smooth muscle cells; VCAM-1, vascular cell adhesion molecule-1, TNF-α: tumor necrosis factor-α; IL-6, Interleukin- 6; ICAM-1, intercellular cell adhesion molecule-1; MCP1, monocyte chemotactic protein-1.

Moreover, other apolipoproteins can contribute to AS by modulating the interaction of ApoB with arterial wall proteoglycans, thereby promoting TRLs remnant accumulation. For instance, ApoE facilitates the binding and retention of ApoB-containing atherogenic lipoproteins with arterial wall glycosaminoglycans, while ApoC-III augments the affinity of TRLs remnants for arterial wall proteoglycans and reduces hepatic uptake of ApoB, thereby exacerbating TRLs remnant accumulation within the arterial wall (56). Abnormal TRLs remnant accumulation prompts phagocytosis by ApoB-induced activated macrophages, eliciting a complex immune-inflammatory response that hastens AS progression (42) (**Figure 2**).

### 4.2 Mechanism of action of LPL for AS development

Disorders in lipid metabolism and inflammatory responses stand as pivotal risk factors in the development and progression of AS. LPL, a crucial enzyme catalyzing TG lipolysis in lipoproteins, is expressed in various tissues including adipose tissue, muscle tissue, and macrophages. Angiopoietin-like protein 3 (ANGPTL3), primarily expressed in the liver and secreted into the bloodstream, plays a significant role in modulating the metabolism of TRLs by inhibiting LPL activity (57–60). ANGPTL3 impacts plasma TRLs levels by influencing their clearance and secretion. It interacts with LPL in circulation, thereby suppressing its activity, which diminishes the breakdown of VLDL-TG and reduces the clearance rate of both VLDL and chylomicrons (58). Additionally, ANGPTL3 promotes adipose tissue catabolism, releasing FFA into circulation, which are then utilized by the liver for VLDL synthesis, further contributing to elevated VLDL-TG secretion into the bloodstream (59, 61). Increased expression or elevated plasma levels of ANGPTL3 exacerbate the inhibition of LPL activity, resulting in the accumulation of pro-atherogenic TRLs in circulation and triggering a cascade of complex inflammatory responses. Human genetic studies have provided evidence suggesting that loss-of-function mutations or downregulation of ANGPTL3 correlate strongly with reduced circulating TG and TRLs levels, consequently reducing the risk of AS (62–64). Consequently, ANGPTL3 has emerged as a promising therapeutic target for mitigating the risk of ASCVD and is under intensive investigation (65). Current research trends are centered on interfering with or inhibiting ANGPTL3 through various approaches, including ANGPTL3 monoclonal antibodies, antisense oligonucleotides (ASO), and gene editing techniques (12, 64, 66).

Evinacumab, a fully human monoclonal antibody featuring a constant region of human IgG4, exhibits high affinity binding to ANGPTL3 across multiple species. It has been demonstrated that REGN1500 can effectively reverse ANGPTL3-induced inhibition of LPL activity both *in vivo* and *in vitro*. Preclinical studies conducted in animals have illustrated that evinacumab enhances LPL activity in both normolipidemic and dyslipidemic mice by inhibiting ANGPTL3, consequently leading to decreased circulating levels of TG, HDL-C, and LDL-C (67). Another animal study similarly suggested that ANGPTL3 monoclonal antibody significantly reduces TG and VLDL levels, as well as the risk of AS, by reducing ApoB-containing proatherogenic lipoproteins (68). Additionally, research indicates that evinacumab may also contribute to the regression of atherosclerotic plaques (69). ASO therapy and small interfering RNA (siRNA) therapy targeting ANGPTL3 mRNA offer promising avenues for intervention. ASOs directed against hepatic ANGPTL3 messenger RNA (mRNA) function by degrading the mRNA and blocking its translation, thereby suppressing ANGPTL3 protein expression (70, 71). Preclinical studies in mice have shown that ANGPTL3 ASO reduces hepatic VLDL-TG secretion, enhances LPL activity, and leads to a significant decrease in circulating TG and TRLs levels, ultimately slowing the progression of AS. Subsequent human trials have validated these findings (72). ARO-ANG3, a siRNA therapeutic, targets hepatocyte ANGPTL3 mRNA to inhibit its synthesis. By upregulating LPL activity and promoting the hydrolysis of TRLs, ARO-ANG3 reduces circulating TG levels and accelerates the clearance of TRLs remnants. Additionally, ARO-ANG3 inhibits hepatic PCSK9 expression, further lowering blood lipid levels. Compared to ANGPTL3 ASO, ARO-ANG3 demonstrates longer efficacy and improved tolerability (73).

Considering the efficacy of ANGPTL3 ASO in managing dyslipidemia, assessing the safety of long-term ANGPTL3 inhibition is crucial. Current evidence suggests that N-acetyl galactosamine (GalNAc)-conjugated ASOs are clinically safe and well-tolerated (73–75). Furthermore, GalNAc-coupled ASO drugs exhibit more significant lipid-lowering effects compared to non-coupled ASO drugs, with only a fraction of the effective dose necessary to achieve comparable reduction in hepatic Angptl3 mRNA levels (72, 76).

In addition to the RNA interference therapies and anti-ANGPTL3 monoclonal antibodies mentioned above, therapeutic gene editing aimed at inducing permanent loss-of-function mutations in ANGPTL3 represents another approach to lowering TG levels and reducing the risk of coronary heart disease (77). *In vivo* ANGPTL3 gene editing has been proposed as a potential treatment strategy for atherosclerotic dyslipidemia (78). However, the safety of *in vivo* gene editing cannot be guaranteed. The introduction of mutant genes may carry risks such as tumorigenesis, irreversible genetic alterations, serious adverse effects, or even therapeutic failure. Therefore, in-depth studies are necessary to thoroughly evaluate the safety and efficacy of *in vivo* gene editing (79).

Furthermore, LPL also plays a role in AS by promoting the accumulation of lipoproteins and triggering inflammatory responses in the vessel wall (29, 80). Primarily, the role of LPL in AS development hinges upon its location. Elevated LPL levels in plasma without concomitant elevation in vessel wall LPL can reduce TG levels and elevate HDL levels. This reduces plasma TRLs concentration and expedites hepatic clearance of residual lipoproteins, thus exerting a protective effect against AS (81). Conversely, within the vessel wall, where macrophages and SMCs express LPL, TRLs remnants accumulate, inciting AS-related responses like lipid accumulation and inflammation (29, 81). LPL facilitates macrophage uptake of small remnants generated by lipolysis, contributing to foam cell formation, a hallmark of early AS (82, 83). Furthermore, vessel wall LPL increases lipoprotein retention in the subendothelial cell matrix and the aorta, suggesting heightened LPL activity in the vessel wall promotes AS progression (29, 81). Secondly, TRLs remnants produced by LPL-mediated lipolysis induce vascular injury, attracting monocyte-derived macrophages to phagocytose accumulated remnants, forming foam cells. Increased endothelial permeability post-vascular injury leads to further TRLs remnant and subendothelial accumulation, which upregulate inflammatory factor and adhesion molecule expression in endothelial cells, promoting monocyte adhesion and reactive oxygen species production, fostering local inflammatory responses (84, 85).

## 5 Lowering TG alone does not provide clinical benefit

Presently, the direct measurement of serum TRLs and TRLs remnants in clinical settings lacks reliable technology. Consequently, serum TG levels are commonly used as an indirect indicator for estimating TRLs and TRLs remnants levels, leading to the development of drugs aiming at reducing TG (35, 86, 87). Fibrates are the primary choice for TG reduction in clinical practice. They specifically bind to and activate peroxisome proliferator-activated receptor alpha (PPARα), increasing LPL activity and certain apolipoproteins, thus enhancing fatty acid oxidation and TRLs clearance. Although fibrates significantly reduce TG levels (by 30%-50%) and non-HDL cholesterol levels (by 6%-16%), previous studies with fenofibrate, benzofibrate, and gefibrate have not shown a reduction in major cardiovascular events (88). However, *post hoc* subgroup analyses revealed benefits in patients with type 2 diabetes mellitus, mild-to-moderate hypertriglyceridemia, and low levels of HDL cholesterol (89, 90).

Pemafibrate, a novel potent PPARα agonist, has shown promising results. It induces LPL expression, reduces serum TG levels, and increases HDL levels. The PROMINENT clinical trial investigated pemafibrate’s potential to reduce cardiovascular events in patients with diabetes and mild-to-moderate elevated TG and reduced HDL-C levels, showing a reduction in TG, VLDL-C, residual cholesterol, and Apo-CIII levels, with no significant difference in the risk of cardiovascular disease compared to the control group (91). This result may be due to the fact that pemafibrate increases the activity of LPL, which leads to an increase in the conversion of TRLs remnants to LDL-C, and the increased LDL-C level may offset the clinical benefits caused by the decrease of TG and VLDL (92).

Another class of drugs used clinically to reduce TG is Omega-3 polyunsaturated fatty acids, with an average of 4 g of Omega-3 polyunsaturated fatty acids per day lowering triglyceride concentrations by an additional 30% from baseline levels (93). The results of the REDUCE-IT study published in 2018 showed that eicosapentaenoic acid (EPA) treatment given to high-risk ASCVD patients with well-controlled LDL-C levels but elevated TG levels after statin treatment reduced serum TG levels by only 2%, ApoB levels (reflecting LDL-C levels) by 9.7% and the major cardiovascular event risk by 25% compared to the placebo group (94). The RESPECT-EPA study published in 2022 evaluated a randomized clinical trial of the effect of combination therapy with statins and EPA for secondary prevention of ASCVD, and similarly achieved significant benefits (95). These results suggest that the cardiovascular protective effects of EPA cannot be explained solely by the reduction of TG, but may also involve other mechanisms including improvement of glucose homeostasis and endothelial cell function, suppression of inflammation levels, oxidative stress and LDL oxidative modification (96). In conclusion, in clinical trials to reduce TG level, simultaneous reduction of TG and LDL-C is beneficial, but lowering TG while elevating LDL-C is not beneficial (96). In conclusion, simultaneous reduction of TG and LDL-C is beneficial, while lowering TG while elevating LDL-C is not advantageous. Currently, no drug significantly reduces TRLs levels without increasing LDL-C levels in clinical practice, posing a significant challenge in lipid-lowering drug development.

## 6 Conclusions

ASCVD remains the leading global cause of mortality, with LDL recognized as a significant atherogenic risk factor. Despite aggressive LDL-lowering therapy, patients still face residual cardiovascular risk. Recent advancements in human genetics, along with epidemiological, preclinical, and clinical trial evidence, strongly indicate a causal link between TG, TRLs, and TRLs remnants, and increased ASCVD risk. Lowering plasma TG levels and reducing TRLs and TRLs remnant production may offer further cardiovascular risk reduction, although this assertion requires reinforcement due to the limited evidence of relevant randomized controlled trials. Fortunately, the role of TG, TRLs, and their remnants in AS development provides a novel and precise therapeutic target for AS treatment. Over the past few years, a variety of promising strategies to lower TG have been developed. However, the aim of reducing atherogenic lipoprotein levels extends beyond mere TG reduction; the cholesterol-rich residue produced by TG lipolysis still carries the risk of promoting AS. Therefore, the challenge of how to achieve TG reduction without concomitant elevation of LDL levels remains to be solved.

## Author contributions

DX: Writing – original draft. LX: Writing – original draft. CC: Writing – review & editing. FX: Writing – review & editing. CS: Writing – review & editing.
